# Farmer-preferred traits in smallholder dairy farming systems in Tanzania

**DOI:** 10.1007/s11250-018-01796-9

**Published:** 2019-02-04

**Authors:** A. R. Chawala, G. Banos, A. Peters, M. G. G. Chagunda

**Affiliations:** 1 SRUC Research, Kings Buildings, West Mains Road, Edinburgh, EH9 3JG UK; 2 Centre for Tropical Livestock Genetics and Health, University of Edinburgh, Easter Bush, Midlothian, EH25 9RG UK; 3 The Roslin Institute, University of Edinburgh, Easter Bush, Midlothian, EH25 9RG UK; 4 Present Address: Department of Animal Breeding and Husbandry in the Tropics and Subtropics, University of Hohenheim, Garbenstr. 17, Stuttgart, 70599 Germany

**Keywords:** Dairy traits, Trait preference, Breeding goal, Choice experiment

## Abstract

Decisions of breeding schemes in many countries in sub-Saharan Africa tend to be either government or project driven, with a focus on upgrading local breeds. However, there is scant information on the individual animal traits that smallholder farmers prefer. The aim of this study was to examine farmers’ preferences of dairy cattle traits using a discrete choice experiment methodology. The study was conducted through visits to 555 randomly selected dairy farms in the sub-humid Eastern coast and temperate Southern highlands of Tanzania. Choices of animal traits were presented to farmers who were asked to evaluate choice alternatives based on attribute levels and finally select the alternative with the highest utility. The choice experiment data were analysed using a conditional logit model. Coefficients for milk yield, fertility, feed requirement, temperament and diseases resistance were overall statistically significant (*p* < 0.05). In order of perceived importance, farmers were willing to keep a cow with high milk yield (coefficient = 1.43 ± 0.059), good fertility (0.85 ± 0.050), easy temperament (0.76 ± 0.066), low feed requirement (− 0.56 ± 0.092) and enhanced tropical disease resistance (0.48 ± 0.048). The purchase price coefficient was negative (− 0.001 ± 0.0003), indicating that farmers would prefer improved dairy cattle at affordable prices. Farmers’ preferred traits were influenced by agro-ecological zone and type of production system (extensive vs intensive). The study provides an opportunity for breeding programme designers to take farmers’ preferred dairy traits into serious consideration.

## Introduction

Smallholder dairy farming, characterised by small herds of 2–3 milking cows, provides a livelihood for more than 150 million farm households worldwide (FAO 2010; DGEA 2015). The majority of smallholder farmers are found in developing countries. In Tanzania, smallholder dairy farming has rapidly developed in the past 3 decades, mainly due to the successful role in poverty alleviation and bridging the gap to increasing demand for milk and milk products. The national dairy cattle herd includes the traditional sector that contributes 70% of the total milk produced, while the remaining amount is produced by the smallholder dairy farmers (Njombe et al. 2011). Studies in dairy marketing show that 90% of the milk produced in the traditional sector is consumed at the household level and only 10% was marketed. This is contrary to smallholder dairy farming where 70% of milk produced was marketed and 30% was consumed at home (Njombe et al. 2011; Katjiuongua and Neglen 2014). Total milk production has increased at a rate of 2.8% per year over the past 20 years as a result of the growth in the cattle population, rather than an increase in productivity per cow, reflecting a rather inefficient milk production system (FAO 2010; Nell et al. 2014; URT 2016). Previous studies have described the mismatch between the genotypes used and the production environment they are kept in as a major cause of poor productivity in the tropics (Mwacharo et al. 2008; Philipsson et al. 2011).

The vision of the Tanzanian livestock industry is to increase animal productivity and commercialise the livestock sector to ensure an improved household food security and income (URT 2006). However, in Tanzania, as in most countries in sub-Saharan Africa, the active participation of smallholder dairy farmers in designing breeding programmes has received little attention (Bebe et al. 2003; Duguma and Janssens 2016). Hence, there is limited information on how farmers contribute to developing and improving the local dairy industry. The International Livestock Research Institute has recently conducted participatory studies—mainly surveys in smallholder dairy production systems—as part of various projects, such as Dairy Genetics East Africa, Tanzania Dairy Genetics, East Africa Dairy Development and More Milk-IT, to identify the important traits that farmers consider when selecting dairy cattle (DGEA 2015). These studies have been mainly based on qualitative research methods, which are limited in revealing trade-offs between traits of interest. Quantitative methods of eliciting preferences such as best-worst scaling and discrete choice experiments have not been used so far in studies aiming to support farmers’ decisions for selecting the best cows in smallholder dairy production systems.

Duguma et al. (2010) and Ndumu et al. (2008) suggested the use of a combination of survey, ranking and choice experiment methods when identifying traits for selection. In trait preference ranking studies, surveys and trait ranking methods are used to collect information at an early stage, with the aim of obtaining a general picture of the list of traits to be considered in a breeding objective. The choice experiment method has been widely used for quantifying farmers’ preference traits for various livestock species, including cattle (Ndumu et al. 2008; Wurzinger et al. 2006), sheep (Ragkos and Abas 2015) and pigs (Roessler et al. 2008). The method quantifies qualitative data to calculate the strength of the preferences and trade-offs, and the probability that attributes will become more widespread. Additionally, this method surpasses both surveys and ranking methods in terms of the ability to elicit preferences and reveal trade-offs among attributes. However, this method has not been used yet to establish the relative importance of farmer-preferred dairy traits in Tanzania.

The aim of the present study was to determine the most important traits preferred by farmers in smallholder dairy farming systems using both qualitative and quantitative methods. The specific objectives were to (1) determine farmers’ preferred dairy traits that could influence the choice of dairy cows; (2) quantify how dairy farmers evaluate cow traits and consider trade-offs between different attributes in the preferred traits; (3) examine differences in farmers’ dairy trait preferences between agro-ecological zones and production systems of Tanzania.

## Materials and methods

### Study areas and survey design

The study was conducted in two agro-ecological zones in Tanzania; the Southern highlands and the Eastern coast. The Southern highland study sites included the Iringa rural, Makambako and Njombe districts, while the sites on the Eastern coast included the Tanga city and Muheza and Korogwe districts. The Eastern coast zone is situated between latitude 4° to 6° S and longitude 37° to 39° E. The study covered the coast belts and lowland areas with an altitude of 0 to 800 m above sea level. Temperatures range from 26 to 32 °C in the hot season (December to March) and 20 to 28 °C temperatures in the cool season of the year. The area receives the amount of 800 to 1400 mm of rainfall a year. The rainfall distribution pattern is bimodal with long and reliable rains starting in February to May and short rain from October to December (URT 1997). In contrast, the Southern highland zone is located at the latitude between 7° to 11.5° S, longitude 30° to 38° E and at an altitude of about 400 to over 2000 m above the sea level. The zone receives a reliable unimodal rainfall distribution pattern ranging from 800 to 3500 mm starting from December to April. The average minimum and maximum temperatures in the highland zone are 10.6 and 26.5 °C, respectively (Bisanda et al. 1998; URT 2016). The two zones were selected based on milk production potential and existence of farmer organisations, milk collection and processing infrastructures. Additionally, there is a considerable variation between the two zones in terms of rainfall patterns, temperature, landscape and socioeconomic activities. Within each region, two production systems were identified: (i) extensive system, characterised by semi or full grazing on natural pastures with limited purchase of inputs and (ii) intensive system, characterised by zero grazing with the increased use of purchased inputs (Ojango et al. 2017). We used a quantitative method (choice experiment) to identify farmers’ preferred dairy traits followed by focused group discussions to test and corroborate choice experiment results.

A choice experiment (CE) questionnaire was used to determine how smallholder dairy farmers view cow traits and how they consider trade-offs between different sets of characteristics (Fishburn 1968). A full factorial CE design with six traits and two levels for each trait was created (Table 1). Thus, a total of 64 (2^6^) combinations of possible choices were available. The final CE design included a total of eight (8) choice sets or questions with three options ‘Alternative 1’, ‘Alternative 2’ and ‘Alternative 3’. Alternatives 1 and 2 included trait combinations for milk yield, fertility, temperament, disease resistance and feed requirement. Alternative 3 corresponded to neither Alternative 1 nor 2. The questionnaire was designed in such a way that each farmer had to respond to a total of eight choice cards.

**Table 1:** Dairy trait and their corresponding levels used in the discrete choice experiment

Attribute	Description	Levels definitions	A priori expectation
i High milk yield	Milk is a source of protein, employment and income for many smallholder dairy farmers. However, there is a vast variation in milk yield between genotypes and production systems. For example, as part of the Dairy Genetics East Africa (DGEA) Project, it was found that higher milk production levels were found in cows under intensive (zero gazing) compared to extensive (grazing and semi-grazing) production systems (DGEA 2015).	Two levels of milk yield of 5 and 10 l/cow/day were chosen, based on the average milk production in semi-intensive and intensive dairy production systems in Tanzania. - Level 1: 5 l/cow/day accounted for the actual milk production per cow of the majority of smallholder dairy farmers (about 90%) (DGEA 2015) - Level 2: 10 l/cow/day accounted for the top 10% of the best smallholder dairy farmers (DGEA 2015)	In general, a positive preference for higher milk yield/cow/day was expected
ii Good fertility	Smallholder dairy farmers are interested in cow fertility to ensure continued milk production on farm. Longer calving interval affects annual milk production and increases labour costs. The reported calving interval of improved dairy cattle in Tanzania ranges between 13 and 16 months, which is comparable to most countries in sub-Saharan Africa.	Two levels were chosen for cow fertility in smallholder dairy farms. - Level 1: one calf after every 1 to 1.25 years, considered good fertility - Level 2: 1 calf after every 2 years, considered poor fertility	In general, a positive preference for cows that produces a calf every year is expected
iii Animals that best convert of the commonly available feeds into milk	Daily feed requirement is important due to seasonal availability of feeds and under developed pastures on most of the farms (DGEA 2015). This attribute describes the adaptability of cows to utilise commonly available feed resources for growth and milk production. Poor growth rate has an impact on age at first calving, feed costs and can reduce lifetime milk production.	Two levels were chosen for the ability of the cow to use the available feed resources - Level 1: smaller body size (low feed requirement) to produce moderate volumes of milk using locally available feed resources - Level 2: large body size (high feed requirements) to produce high volumes of milk using locally available feed resources	Current breeding strategies are based on increased milk volumes per cow. Therefore, a positive preference for cows with higher milk volumes and hence higher feed requirements was expected.
iv Temperament	Good temperament is used as criteria for easy handling of cows. During the focused group discussions; temperament was a prominent trait for farmers under semi-grazing systems where animals were taken for grazing or tethered in pasture plots.	Preference for temperament was assessed in two levels. - Level 1: docile cow/easy to care - Level 2: aggressive cow	In general, a positive preference for good temperament of cows was expected.
v Animals better adapted to the local production environment.	This attribute includes a range of climatic factors affecting cow productivity. Adaptability to temperature and diseases affects the economic performance of a cow directly through reduced veterinary costs and improved quality of products. For example, in coastal areas, adaptability to hot and humid coastal environments such as tolerance to high ambient temperatures and high humidity are essential. In the highlands and medium altitude areas, ability to cope with different disease, e.g. tick-borne diseases is important.	Two levels of adaptability to production environment were chosen based on annual disease incidences and of use of veterinary services. - Level 1: animal frequently treated for various diseases treatments (> = 4 times a year) - Level 2: animal rarely treated for various diseases (< 4 times a year)	In general, a positive preference for low incidence of veterinary service use was expected
i Purchase price of cow with the desired traits	The purchase price attribute was based on the current prevailing market price for dairy heifers in Tanzania. For example, as part of the DGEA project in Tanzania, it was reported that purebred animals fetched a higher price than crossbreds. The price range for improved dairy cows in Tanzania ranges between 750,000–1,200,000 TZS with an average of 850,000 TZS (DGEA 2015).	Two price levels were included based on current market prices for improved dairy cattle in Tanzania. - Level 1: 750,000 TZS—equivalent to £250. This accounted for a lower price for improved dairy cattle. - Level 2: 1,200,000—equivalent to £400. This accounted for a higher price for improved dairy cattle.	Positive preference for reduced animal price was expected

### Data collection and statistical analysis

A purposive sample of five wards per district was selected for a study based on the prior information on wards participating in the data-recording scheme under the African Dairy Genetic Gains Project (ILRI 2017). In each ward, 15 smallholder dairy farmers were randomly chosen from a list of households that were participating in data recording as part of the African Dairy Genetic Gains project. The minimum sample size required for each zone was calculated by the following equation suggested by Orme 2010.

$$ N\ge 50 0c/\left(t\times a\right) $$

Where *N* is the number of respondents, ‘*c*’ is the largest number levels for any trait, ‘*t*’ is the number of choice tasks and ‘*a*’ is the number of alternatives per task. This sample of households was selected from about 2000 households in the two regions that were participating in the data recording as part of the African Dairy Genetic Gains project (ILRI 2017).

A total of 286 and 269 households were selected on the Southern highland and Eastern coast, respectively. The number of households sampled in extensive and intensive systems was 131 and 424, respectively, across the two regions. Choices of animal traits were presented to the farmers who were then asked to evaluate choice alternatives based on attribute levels and finally select the alternative with the highest utility.

Data were analysed using the following conditional logit model; the model was applied both within and across agro-ecological regions and production systems:

Model $$ \Pr \left(\mathrm{Choice}\right)={\beta}_0+\sum \limits_i{\beta}_i{X}_i+{\varepsilon}_{\mathrm{i}} $$

Where Pr (Choice) is the probability of choosing a specific trait combination; *β*_0_ is the alternative specific constant (intercept); *β*_*i*_ is the reference weight for trait *i*; *X*_*i*_ is the level of trait *i*; *ε*_*i*_ is the error term*.*

Solutions for the *β*_*i*_ values corresponded to estimate coefficients of farmer-preferred dairy traits. Higher estimates of the coefficients corresponded to higher emphasis placed on this trait by the farmer.

The goodness of fit of the model was measured by the likelihood ratio test (*ρ*^2^ is pseudo rho-squared) defined as (McFadden 1977):

$$ \rho 2=1-\frac{\mathrm{Log}\ \mathrm{likelihood}\ \mathrm{of}\ \mathrm{model}}{\mathrm{Log}\ \mathrm{likelihood}\ \mathrm{of}\ \mathrm{model}\ \mathrm{without}\ \mathrm{predictors}} $$

Marginal willingness to pay (MWTP) for each trait was estimated as follows:

$$ \mathrm{MWTP}=-{1}^{\ast }\ \left(\frac{\beta_i}{\beta_{\mathrm{price}}}\right) $$

Where *β*_*i*_ is the estimated coefficient of trait *i*, *β*_price_ is the estimated price coefficient (Roessler et al. 2008; Aizaki 2012). The 95% confidence intervals for MWTP were estimated using a simulation method, as proposed by Krinsky and Robb (1986) with 10,000 replications. The MWTP for traits was calculated by agro-ecological zones to account for the variation in a farmer’s preference traits, climatic factors and milk marketing strategies.

The functions *clogit*, *gofm* and *mwtp* of “survival and support.CEs packages” (Aizaki 2012; Therneau 2015) R (R Core Team 2017) software were used to estimate the above coefficients, goodness of fit of the fitted models and MWTP, respectively.

## Results

The overall results across both agro-ecological zones and production systems are presented in Table 2. Coefficients for animal milk yield, fertility, feed requirement, temperament and disease resistance traits were statistically significant (*p* < 0.05). Results indicate that, overall, farmers were willing to keep a cow with high milk yield, good fertility, easy temperament, low feed requirement and high tropical disease resistance, in order of importance. The purchase price coefficient was negative, indicating that farmers prefer to pay less for improved dairy cows. The overall fit of the model was considered good, with a pseudo *ρ*^2^ of 0.30. According to McFadden (1977), *ρ*^2^ values ranging from 0.2 to 0.4 indicate a good model fit.

**Table 2:** Estimates of overall dairy preference traits for smallholder dairy farmers in Tanzania

	Coefficient ± SE	*p* value
Intercept	0.85 ± 0.159	< 0.0001
Milk yield	1.43 ± 0.059	< 0.0001
Fertility	0.85 ± 0.050	< 0.0001
Feed requirement	− 0.56 ± 0.092	< 0.0001
Temperament	0.76 ± 0.066	< 0.0001
Disease resistance	0.48 ± 0.048	< 0.0001
Price	− 0.001 ± 0.0003	0.0004
Rho-squared	0.30	
Number of observations	13,320	
LL of the model	− 3399.21	
LL of the model without predictors	− 4842.68	

*LL*, log likelihood; *SE*, standard error

The coefficients for the same traits chosen by farmers within different agro-ecological zones and dairy production systems are presented in Table 3. Coefficients for all traits were significant (*p* < 0.05) for farmers in intensive production systems in both the Southern highland and Eastern coastal zones. Regarding the extensive production system, only the coefficient for disease resistance was significant in the Southern highland zone, whereas, all trait coefficients except for feed requirement were significant in the Eastern coastal zone. The price coefficient was only significant (*p* < 0.05) for the intensive dairy production system in the Eastern coastal zone. Moderate to high model fit values (0.33–0.37) were observed except for the Southern highland extensive production system, which was characterised by the lowest amount of available data.

**Table 3:** Estimates of smallholder farmers’ preference traits in two agro-ecological zones and production systems

	Southern highland zone				Eastern coastal zone			
	Intensive systems		Extensive system		Intensive systems		Extensive system	
	Coefficient ± SE	*p* value	Coefficient ± SE	*p* value	Coefficient ± SE	*p* value	Coefficient ± SE	*p* value
Intercept	0.04 ± 0.247	0.85	1.74 ± 0.718	0.016	2.88 ± 0.343	< 0.0001	0.03 ± 0.345	0.92
Milk yield	2.16 ± 0.088	< 0.0001	0.12 ± 0.295	0.691	0.78 ± 0.126	< 0.0001	1.14 ± 0.128	< 0.0001
Fertility	0.82 ± 0.075	< 0.0001	0.12 ± 0.242	0.627	0.81 ± 0.093	< 0.0001	0.98 ± 0.129	< 0.0001
Feed requirement	− 0.59 ± 0.138	< 0.0001	− 0.04 ± 0.424	0.925	− 0.59 ± 0.199	0.0028	− 0.09 ± 0.205	0.65
Temperament	0.70 ± 0.095	< 0.0001	0.12 ± 0.305	0.700	0.36 ± 0.12	0.0046	1.66 ± 0.178	< 0.0001
Disease resistance	0.52 ± 0.077	< 0.0001	0.52 ± 0.235	0.026	0.36 ± 0.092	< 0.0001	0.72 ± 0.111	< 0.0001
Price	− 0.001 ± 0.0005	0.088	− 0.002 ± 0.0016	0.270	− 0.002 ± 0.0006	0.036	− 0.001 ± 0.0008	0.18
Rho-squared	0.37		0.18		0.33		0.33	
Number of observations	6432		432		3744		2712	
LL of the model	− 2326.86		− 129.74		− 910.50		− 666.53	
LL of the model without predictors	− 1465.04		− 158.20		− 1364.48		− 993.15	

*LL*, Log Likelihood; *SE*, Standard error

Table 4 presents the estimates of the marginal willingness of the farmers to pay (MWTP) and the coefficients for their preferred traits in the Southern highland and Eastern coast. Milk yield, fertility, temperament and disease resistance had a positive MWTP, while feed requirement had negative MWTP values in both agro-ecological zones. The MWTP estimates show the amount of money farmers are willing to pay for dairy cattle possessing traits of their interest on top or less to what they normally pay. In both agro-ecological zones, farmers were willing to invest for cows with high milk production, good fertility, easy temperament and high disease resistance. Conversely, farmers were willing to pay less for cows with high feed requirements. The confidence intervals for these values were high, demonstrating the magnitude of farmer variability in willingness to pay for cows with the desired characteristics.

**Table 4:** Farmer marginal willingness to pay and preferences for each trait by agro-ecological zone

	Southern highland zone				Eastern coastal zone			
	^1^MWTP (£)	MWTP (£) 95% CI	Coefficient ± SE	*p* value	MWTP (£)	MWTP (£) 95% CI	Coefficient ± SE	*p* value
Intercept			1.17 ± 0.712	0.100			2.73 ± 0.677	< 0.0001
Milk yield	404.42	172.05–2358.33	2.01 ± 0.083	< 0.0001	47.33	85.26–465.20	0.89 ± 0.087	< 0.0001
Fertility	156.02	57.99–944.66	0.77 ± 0.071	< 0.0001	147.34	76.93–517.02	0.89 ± 0.074	< 0.0001
Feed requirement	− 115.08	(− 581.18)–(− 42.07)	− 0.57 ± 0.130	< 0.0001	− 71.43	(− 187.59)–(− 31.48)	− 0.43 ± 0.138	0.0018
Temperament	134.93	44.94–826.51	0.67 ± 0.090	< 0.0001	138.70	64.82–520.97	0.83 ± 0.100	< 0.0001
Disease resistance	102.60	38.18–573.79	0.51 ± 0.072	< 0.0001	74.64	40.63–226.90	0.45 ± 0.068	< 0.0001
Price			− 0.005 ± 0.0025	0.005			− 0.006 ± 0.0023	0.0094
Rho-squared			0.34				0.31	
Number of observations			6864				6456	
LL of the model			− 2485.06				− 2357.62	
LL of the model without predictors			− 1629.07				− 1638.42	

*LL*, log likelihood; *SE*, standard error; *CI*, confidence interval; ^1^*MWTP*, marginal willingness to pay; £, British pound sterling

Currency exchange rate: 1 British pound = 2956.04 Tanzanian shillings (31 July 2017 https://www.xe.com)

## Discussion

### Farmers’ preferences and trade-offs of dairy cattle traits across agro-ecological zones and production systems

Results from the analyses across zones and production systems revealed the highest preferences for improved animal production (milk yield and fertility), welfare (good temperament) and adaptability (low feed requirement and tropical disease resistance) traits. Farmers were sensitive to high costs and suggested they would like to acquire improved dairy cattle at an affordable price.

Milk production had the highest positive significant coefficient, indicating that, above all, farmers would like cows with high genetic potential for increased milk yield. More emphasis on production traits compared to other traits indicates the priority of continued milk production for household income, as reported in previous studies (Swai and Karimuribo 2011; Gillah et al. 2014). The farmers’ preference towards high milk production could be associated with an increase in the per capita income and favourable policies towards increased milk production capacity and dairy product safety standards (URT 2013). A similar higher preference for cows with high milk production was previously reported in smallholder dairy farmers in Ethiopia (Duguma and Janssens 2016) and Kenya (Kariuki et al. 2017). Additionally, the preference for high yielding cows could be partly influenced by the existing multiple milk marketing channels. Previous reports showed that 70% of the milk produced in Tanzania reaches the consumer via informal milk marketing channels, also known as ‘milk hawkers’. The remaining 30% of the milk produced reaches consumers through conventional marketing channels via milk collection centres linked to dairy processors (URT 2016).

Fertility had a positive significant coefficient, indicating that farmers preferred productive animals to ensure a continued supply of milk. A profitable dairy enterprise depends on lifetime milk production and thus regular calvings. Fertility is broadly affected by the interaction of genetic and non-genetic factors (De Kruif 1978). Previous studies in Tanzania showed that both unreliable natural mating practices and poor artificial insemination services have led to long intervals between calving, long day open periods and a high number of services per conception (Msangi et al. 2005; Kivaria et al. 2006). Additionally, fertility might be affected by factors such as seasonal feed availability, climatic conditions, disease incidence and management practices (Mwatawala et al. 2002).

Our results showed that smallholder farmers preferred keeping cows with an easy temperament, as the third most important criterion after increased milk yield and fertility. Temperament was defined as the level of cow aggressiveness during handling or milking. Previous studies involving smallholder dairy farmers in Tanzania, Kenya, Uganda and Ethiopia have reported an easy temperament as an important trait when selecting dairy cattle (DGEA 2015). Preference for an easy temperament is associated with the use of family labour in feeding, milking, health management and breeding of dairy cattle. During the focused group discussion, farmers commented on the active participation of women and children in feeding cows as the main reason for disliking cows with aggressive behaviour. Thus, to ensure continued interest in dairy production and possible adoption of newly improved breeds, traits such as docility need to be considered in breeding scheme designs for smallholder dairy farmers.

Importantly, our results showed that most farmers preferred cows with low feed requirements for growth and milk production. Farmers were not willing to acquire cows that required high feed input to produce milk. Negative preference towards cows with a high feed demand and uptake can be related to the cut-and-carry feeding system, which is labour intensive. Preference for breeds with lower feed requirements could be associated with seasonal feed availability. Land shortage for pasture establishment and poor quantity and quality of forages during the dry season have been perceived as major constraints to the dairying activities in Tanzania (Kavana et al. 2005).

Another important trait used for selecting cows was resistance to tropical diseases. The coefficient for disease resistance was positive and highly significant, indicating that farmers’ preferred cows that can withstand tropical disease challenges. Animal diseases are among the factors reported to affect the smallholder dairy sector in Tanzania. Common diseases which affect dairy cattle reported in previous studies include tick-borne diseases, mastitis, contagious bovine pleuropneumonia, foot-and-mouth disease, trypanosomosis, helminthiasis and zoonotic diseases such as brucellosis and bovine tuberculosis (Swai et al. 2010; Karimuribo et al. 2006; MALF 2016). Poor utilisation of extension services, high cost of drugs, low adoption of vaccination programmes and poor disease-reporting systems are among the factors contributing to high incidence of diseases (MALF 2016).

Cow purchase price was considered one of the most important factors when selecting the preferred cow. The price coefficient was negative and highly significant, indicating that farmers preferred dairy cattle purchased at a low price. High prices and lack of market information of improved heifers have been reported as a major constraint in acquiring improved dairy cattle. The purchase price tends to be affected by breed type and production environment (DGEA 2015). Our results suggest that smallholder dairy farmers would benefit from a government policy ensuring an affordable price for improved animals. Therefore, the present study highlights the need for enhancing government policies, technologies and innovations to produce affordable improved dairy cattle for smallholder dairy farmers in Eastern Africa.

### Farmers’ preferences and trade-offs of dairy cattle traits in different agro-ecological zones and production systems

Despite being ranked relatively low in overall preference, disease resistance was the only animal trait viewed as significantly important among farmers in both agro-ecological zones and production systems. Otherwise, there were variations in the patterns described above. Thus, dairy farmers in intensive production systems in both agro-ecological zones indicated a high preference for cows with low feed requirements, which was not the case among their counterparts in extensive production systems. Furthermore, preferences for milk yield, fertility and temperament traits were specific to different agro-ecological zones and production systems. The variation of farmer preference traits across agro-ecological zones and production systems could be associated with environmental factors (availability of feed, disease prevalence) as well as infrastructure (e.g. milk marketing strategies and reliability of breeding services).

Animal production, welfare and adaptability traits were considered of the greatest importance for Southern highland intensive production systems. The highland regions are cooler and, therefore, have a more suitable climate for crossbred and purebred dairy cattle. Generally, dairy farm intensification occurs more rapidly in the highland zones due to small farm sizes for forage establishment (Swai and Karimuribo 2011). In addition, urban and peri-urban dairy farming by-laws advocate intensive feeding systems and a limited number of cattle per household. It could, therefore, be argued that favourable climatic conditions, production systems and local government by-laws have an impact on farmer preference traits.

The difference in farmer trait preference was evident between intensive and extensive systems for farmers in the Eastern coastal zone. In intensive husbandry systems, milk yield, fertility and low feed requirement were the top three most important traits preferred by smallholder dairy farmers. Preference for high production and adaptability traits in these areas is thought to be influenced by the Tanga Dairy Cooperative Union and Tanga Fresh Factory which is the main milk buyer. The Dairy Cooperative Union and milk-processing factory provide inputs, milk collection facilities and a reliable market for the produced milk.

In conclusion, from a farmer’s viewpoint, the most important dairy traits included high milk yield, good fertility, easy temperament, low feed requirements and disease resistance. Farmers’ trait preferences differed between agro-ecological zones and production systems due to a variation of climatic conditions, feed resources and local infrastructure. Thus, adaptability to the local environment was considered as a fundamental trait for selecting dairy cattle. Farmers were willing to invest in improved dairy cattle showing desired traits at an affordable price. Results from the present study provide evidence for designers of breeding programmes to take consideration of specific farmers’ preferred traits. Selection indexes and breeding strategies need to be developed based on the identified farmer preferred traits in the specific agro-ecological zones and production systems.

## References

[CR1] Aizaki H (2012). Basic Functions for Supporting an Implementation of Choice Experiments in R. Journal of Statistical Software, Code Snippets.

[CR2] Bebe BO, Udo HMJ, Rowlands GJ, Thorpe W (2003). Smallholder dairy systems in the Kenya highlands: Cattle population dynamics under increasing intensification. Livestock Production Science.

[CR3] Bisanda, S., W. Mwangi, H. Verkuijl, A.J. Moshi, and P. Anandajayasekeram. 1998. Adoption of Maize Production Technologies in the Southern Highlands of Tanzania. Mexico, D.F.: International Maize and Wheat Improvement Center (CIMMYT), the United Republic of Tanzania, and the Southern Africa Centre for Cooperation in Agricultural Research (SACCAR)

[CR4] De Kruif A (1978). Factors influencing the fertility of a cattle population. Journal of Reproduction and Fertility.

[CR5] DGEA, 2015. Dairy Genetics East Africa. Project Report. Dairy Farm Baseline Survey Report for Tanzania and Ethiopia https://ilri-angr.wikispaces.com/DGEA%201. Accessed 22 March 2017

[CR6] Duguma B, Janssens GPJ (2016). Assessment of feed resources, feeding practices and coping strategies to feed scarcity by smallholder urban dairy producers in Jimma town, Ethiopia. SpringerPlus.

[CR7] Duguma, G., Mirkena, T. Haile, A. Iñiguez, L. Okeyo, A. M. Tibbo, M. and Wurzinger, M., 2010. Participatory approaches to investigate breeding objectives of livestock keepers. Livestock Research for Rural Development, 22, Article #64

[CR8] FAO, 2010. Status and Prospects for Smallholder Milk Production: A Global Perspective, by T. Hemme and J. Otte. Rome. http://www.fao.org/docrep/012/i1522e/i1522e.pdf. Accessed 15 February 2018

[CR9] Fishburn, P.C., 1968. Utility theory. Management Science: Theory Series (Jan. 1968), 14, 335–378

[CR10] Gillah KA, Kifaro GC, Madsen J (2014). Effects of management practices on yield and quality of milk from smallholder dairy units in urban and peri-urban Morogoro, Tanzania. Tropical Animal Health and Production.

[CR11] ILRI, 2017. International Livestock Research Institute Communications. African Dairy Genetic Gains. https://africadgg.wordpress.com/2017/03/27/outcomes/. Accesed 20 May 2017

[CR12] Karimuribo ED, Fitzpatrick JL, Bell CE, Swai ES, Kambarage DM, Ogden NH, French NP (2006). Clinical and subclinical mastitis in smallholder dairy farms in Tanzania: Risk, intervention and knowledge transfer. Preventive Veterinary Medicine.

[CR13] Kariuki C, van Arendonk J, Kahi A, Komen H (2017). Multiple criteria decision-making process to derive consensus desired genetic gains for a dairy cattle breeding objective for diverse production systems. Journal of Dairy Science.

[CR14] Katjiuongua, H. and Nelgen, S., 2014. Tanzania smallholder dairy value chain development: Situation analysis and trends ILRI Project Report. Nairobi, Kenya: ILRI. pp 39. https://cgspace.cgiar.org/bitstream/handle/10568/68513/PR_Tanzania.pdf?sequence=1. Accessed 24 March 2017

[CR15] Kavana, P. Y., Kizima, J. B. Msanga, Y. N. Kilongozi, N. B. Msangi, B. S. J. Kadeng’uka, L. A. and Shimba, P. K., 2005. Potential of pasture and forage for ruminant production in Eastern zone of Tanzania. Livestock Research for Rural Development, 17, Article#144

[CR16] Kivaria FM, Noordhuizen JPTM, Kapaga AM (2006). Prospects and constraints of smallholder dairy husbandry in the Dar es Salaam region, Tanzania. Outlook on Agriculture.

[CR17] Krinsky I, Robb AL (1986). On Approximating the Statistical Properties of Elasticities. The Review of Economics and Statistics.

[CR18] MALF, 2016. Ministry of Agriculture, Livestock and Fisheries. The Tanzania Smallholder Livestock Sector: A review based on the 2012/2013 National Panel Survey. http://www.mifugouvuvi.go.tz. Accessed 3 April 2018

[CR19] McFadden, D., 1977. Quantitative Methods for Analyzing Travel Behaviour of Individuals: Some Recent Developments. Cowles Foundation Discussion Papers 474. In D. Hensher and P. Stopher (eds.), Behavioural Travel Modelling, Croom Helm, 1978, 279–318

[CR20] Msangi BSJ, Bryant MJ, Thorne PJ (2005). Some factors affecting variation in milk yield in crossbred dairy cows on smallholder farms in north-east Tanzania. Tropical Animal Health and Production.

[CR21] Mwacharo, J. M., Ojango, J. M. K. Baltenweck, I. Wright, I. Staal, S. Rege, J. E. O. and Okeyo, A. M., 2008. Livestock Productivity Constraints and Opportunities for Investment in Science and Technology. BMGF-ILRI Project on Livestock Knowledge Generation. https://cgspace.cgiar.org. Accesed 18 February 2018

[CR22] Mwatawala HW, Kifaro GC, Petersen P (2002). Genetic and Phenotypic Parameters of Reproduction and Lactation Traits of Friesian X Boran Crossbred cattle in Kagera Region, Tanzania. Tanzania Journal of Agricultural Science.

[CR23] Ndumu DB, Baumung R, Wurzinger M, Drucker AG, Okeyo AM, Semambo D, Sölkner J (2008). Performance and fitness traits versus phenotypic appearance in the African Ankole Longhorn cattle: A novel approach to identify selection criteria for indigenous breeds. Livestock Science.

[CR24] Nell, J.A., Schiere, H. and Bol, S., 2014. Quick Scan Dairy Sector Tanzania. http://www.best-dialogue.org. Accessed 13 November 2016

[CR25] Njombe, A.P., Msanga, Y., Mbwambo, N., Makembe, N., 2011. United Republic of Tanzania Ministry of Livestock and Fisheries Development the Tanzania Dairy Industry : Status, Opportunities and Prospects. In 7th African Dairy Conference and Exhibition, MovenPick Palm Hotel, 25-27 May 20111. pp. 25–27

[CR26] Ojango JMK, Mrode R, Okeyo AM, Rege JEO, Chagunda MGG, Kugonza DR, van Belzen N (2017). Achieving sustainable production of milk. Part 3: Improving smallholder dairy farming in Africa.

[CR27] Orme B (2010). Getting Started with Conjoint Analysis: Strategies for Product Design and Pricing Research. Chapter 7. Sample Size Issues for Conjoint Analysis.

[CR28] Philipsson, J., Rege, J. Zonabend, E. and Okeyo, A., 2011. Sustainable breeding programmes for tropical low-and medium input farming systems. Sustainable Breeding Programmes for Tropical Farming Systems In: Animal Genetics Training Resource, Version 3, 2011. Ojango, J.M., Malmfors, B. and Okeyo, A.M. (Eds). International Livestock Research Institute, Nairobi, Kenya, and Swedish University of Agriculture, pp 1–35

[CR29] R Core Team (2017). R: A language and environment for statistical computing. R Foundation for Statistical Computing, Vienna, Austria.URL https://www.R-project.org/. Accessed 24 November 2017.

[CR30] Ragkos A, Abas Z (2015). Using the choice experiment method in the design of breeding goals in dairy sheep. Animal.

[CR31] Roessler R, Drucker AG, Scarpa R, Markemann A, Lemke U, Thuy LT, Valle Zárate A (2008). Using choice experiments to assess smallholder farmers’ preferences for pig breeding traits in different production systems in North–West Vietnam. Ecological Economics.

[CR32] Swai E, Karimuribo E (2011). Smallholder dairy farming in Tanzania: Current profiles and prospects for development. Outlook on Agriculture.

[CR33] Swai ES, Karimuribo ED, Kambarage DM (2010). Risk factors for smallholder dairy cattle mortality in Tanzania. Journal of the South African Veterinary Association.

[CR34] Therneau, T., 2015. R: A Package for Survival Analysis in S_. version 2.38. Available at: url: https://CRAN.R-project.org/package=survival. Accessed 29 March 2018

[CR35] URT, 1997. United Republic of Tanzania. Tanga Region Socio-economic profile. The Planning Commission Dar es salaam and Regional Commissioner Office Tanga. Retrieved from http://www.tzonline.org/pdf/Tanga.pdf. Accesed 19 November 2018

[CR36] URT, 2006. United Republic of Tanzania. Ministry of Livestock and Fisheries Development. National Livestock Policy. http://www.tnrf.org. Accesed 26 March 2018, 11–12

[CR37] URT, 2013. United Republic of Tanzania. Ministry of Livestock and Fisheries Development. Livestock Development Strategy. http://www.tanzania.go.tz. Accesed 23 January 2018

[CR38] URT, 2016. United Republic of Tanzania. Southern Highlands Milkshed Development Project: Project No. 2000001143: Report No: 4240-TZ, East and Southern Africa Division. https://webapps.ifad.org . Accesed 26 March 2018

[CR39] Wurzinger, M., Ndumu, D. Baumung, R. Drucker, A. G. Okeyo, A. M. Semambo, D. K. and Sölkner, J., 2006. Assessing stated preferences through the use of choice experiments: valuing (re)production versus aesthetics in the breeding goals of Ugandan Ankole cattle breeders. In Proceedings of the 8th World Congress on Genetics Applied to Livestock Production 2006,( Belo Horizonte, Minas Gerais, Brazil, Instituto Prociência), 31–39

